# Dietary Supplementation With Yeast Cell Wall Modulates Gut Microbiota and SCFAs Production to Improve Intestinal Health in Adult Cats

**DOI:** 10.1002/fsn3.71007

**Published:** 2025-09-26

**Authors:** Xiaoxi Liu, Haotian Wang, Yuqing Lu, Guoqing Jin, Le Fu, Hongxia Mao, Yi Wu, Pan Liu

**Affiliations:** ^1^ State Key Laboratory of Animal Nutrition and Feeding, College of Animal Science and Technology China Agricultural University Beijing China; ^2^ Department of Animal Science, College of Agriculture and Environmental Science University of California Davis California USA; ^3^ Lanse Maitian Technology (Shanghai) Co. Ltd Shanghai China

**Keywords:** adult cats, gut microbiota, intestinal barrier, intestinal inflammation, scfas, yeast cell wall

## Abstract

Yeast cell wall (YCW) has shown several probiotic activities, including modifying intestinal flora structure, decreasing the abundance of pathogenic bacteria, and regulating intestinal morphology. However, the effect of dietary addition of YCW on intestinal health in cats has not been fully studied. In this study, 20 healthy adult cats were split into a control group (CON) and a YCW addition group (YCW) for a 42‐day trial. On Day 21 of the experiment, the fecal Calprotectin level in the YCW group was significantly lower than that in the control group (*p* < 0.05). By measuring the content of short‐chain fatty acids (SCFAs), we found that YCW supplementation elevated the propionate level in cats' feces (*p* < 0.05). On Day 42 of the experiment, the serum levels of interleukin‐1β (IL‐1β) and diamine oxidase (DAO), as well as the fecal level of Calprotectin, were markedly declined in the YCW group when compared to the control group (*p* < 0.05). We further discovered that the YCW treatment significantly enriched several intestinal beneficial genera in cats, such as Bacteroidaceae, Ruminococcaceae, *Blautia*, and *Lachnoclostridium* (*p* < 0.05). Additionally, cats in the YCW group showed higher levels of acetate and propionate (*p* < 0.05). This study suggested that ingestion of YCW could relieve intestinal inflammation and enhance intestinal barrier function in cats, which may be related to its capacity to enhance the gut microbiota composition and the SCFAs concentrations.

## Graphical abstract

Incorporating YCW into the food of cats improved the intestinal barrier function and alleviated intestinal inflammation. Moreover, dietary YCW increased the percentage of Bacteroidaceae, Ruminococcaceae, *Blautia*, and *Lachnoclostridium*, and elevated the contents of metabolites such as acetate and propionate, which might be responsible for the greater intestinal barrier and immunity function.

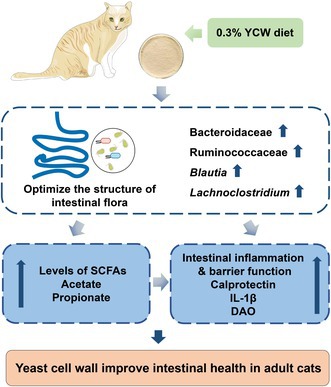

## 1 Introduction

An increasing number of families keep pets, and pet owners around the world are increasingly viewing dogs and cats as members of the family or companions, which is indicative of societal advancement and the enhancement of people's quality of life. The health of dogs and cats, especially the relationship between nutrition and intestinal health, has received widespread attention.

The application of prebiotics is a promising approach to selectively stimulate the activity and growth of beneficial bacteria present in the intestinal ecosystem, which thereby promotes the host health (Sanders et al. 2019). Most of the identified prebiotics are carbohydrates with different molecular structures that are present in the diets of animals and humans. Given that digestive enzymes cannot break down prebiotics, they enter the large intestine in their original form and are fermented by the gut bacteria (Quigley 2019). The effect of prebiotics on the composition and metabolic activity of the gut microbiota largely depends on their fermentative capability. The end products of prebiotic fermentation are mostly short‐chain fatty acids (SCFAs), primarily acetate, butyrate, and propionate, which are then used as energy sources by the host intestinal epithelial cells (Ashaolu et al. 2021). Appropriate SCFAs promote the host health by influencing the immune system, controlling lipid metabolism, and altering intestinal function. Some bacteria, such as *Bifidobacterium* or *Lactobacillus*, produce active compounds that inhibit the development of gastrointestinal pathogens during the fermentation of prebiotics, as well as result in a decline in intestinal pH (Suez et al. 2019).

Yeast or yeast‐derived nutritional supplements have been used in animal feed and human food production, and have shown a variety of biological activities such as improving intestinal flora structure, reducing intestinal barrier permeability, and enhancing body immunity (Bass et al. 2019). Yeast feed additives are commercially available in various forms, including live yeast, yeast cultures, yeast cell walls (YCWs), and their components. YCW is a prebiotic mainly composed of α‐D‐mannan and β‐D‐glucan, which has a positive impact on animal health by creating favorable conditions for helpful microorganisms, lowering the abundance of harmful bacteria, and regulating intestinal morphology (M'Sadeq et al. 2015). According to prior research, 0.05% YCW supplementation increased the gene expression of intestinal tight junction proteins and may be linked to the optimal intestinal morphology and integrity in broilers (Kyoung et al. 2023). Another study demonstrated that weaned pigs fed YCW had lower diarrhea frequency and higher apparent ileal digestibility of dry matter, and dietary YCW could modify immune responses of weaned pigs by decreasing serum contents of tumor necrosis factor‐α (TNF‐α) and interleukin‐1β (IL‐1β) (Lee et al. 2021). In addition, a study examined how a supplement containing YCW fraction affected the gut health and fecal features of adult dogs following a sudden change in diet. It found that the YCW fraction contributed to increased levels of *Clostridium perfringens* and IgA in the feces of the adult dogs (Lin et al. 2020).

Although cats are natural carnivores and have a different gut microbiota structure than omnivores and herbivores, they still harbor a large number of microbes in their hindgut (Alessandri et al. 2020). Numerous studies have shown that dietary modifications to the gut microbiota and its metabolites are of great significance to the overall health of felines (Lee et al. 2022; Mo et al. 2023; Wang et al. 2023). Since the existing studies mainly focus on economic animals, conducting experiments to explore how YCW affects the intestinal health of cats not only can promote the theoretical development of cat nutrition, but also help to reveal the mechanism of the nutritional‐microbial interaction of carnivorous hosts. A former investigation has assessed the influence of adding a mixture of YCW components to cat food on their fecal microbiota and fermentation reactions both in vivo and in vitro, and found that YCW could reduce the fecal pH, regulate the fecal microbiota, and affect the fecal SCFAs production (González et al. 2023). However, their research primarily concentrated on describing shifts in microbial communities and basic fermentation parameters, without extensively clarifying the functional implications of these changes for host intestinal health, particularly in terms of immune regulation and intestinal barrier integrity. Therefore, in order to shed light on the future development and use of YCW in pet feed, this study sought to examine the effects of dietary YCW supplementation on the intestinal microbial structure, immunological function, and intestinal health in cats.

## 2 Materials and Methods

### 2.1 Materials

YCW powder was purchased from DOCHOO Biotechnology Co. Ltd. (Zhejiang, China), and the component analysis of YCW was shown in the Table S1. Enzyme‐linked immunosorbent assay (ELISA) kits for IL‐6, TNF‐α, IL‐1β, lipopolysaccharide (LPS), Calprotectin, alpha‐1 antitrypsin (α1‐AT), diamine oxidase (DAO), Zonulin, and intestinal fatty acid‐binding protein (I‐FABP) were obtained from Mlbio Co. LTD (Shanghai, China).

### 2.2 Methods

#### 2.2.1 Ethics Statement

The China Agricultural University Institutional Animal Care and Use Committee gave its approval to the experimental protocol, and the license code was AW07254202‐2‐1.

#### 2.2.2 Animals and Experimental Design

The following criteria were applied to choose the experimental cats. All cats were 1–3 years old and had normal body condition scores (BCS). No immune‐mediated disorders, allergies, or other conditions that cause chronic gastrointestinal dysfunction, such as pancreatic insufficiency, liver disease, metabolic disease, renal disease, or parasite disease, were present in any of the cats. The animals had not been administered any medication or feed related to the tested functions for 3 months prior to the study. The animals had not been treated with antibiotics or immunosuppressive drugs and had not undergone surgical procedures. In addition, pregnant or lactating animals and animals that cannot eat orally were excluded from the study.

Twenty adult cats were selected for this experiment, half male and half female. There were 2 treatment groups with 10 replicates per group and 1 cat per replicate: (1) the control group (CON): cats were fed with the basal diet; (2) the YCW addition group: cats were fed with the diet supplemented with 0.3% YCW. YCW was added during the mixing process of raw materials and was not subjected to a high‐temperature puffing treatment. The formal trial lasted for 42 days. On Day 42 of the experiment, 3–5 g of fresh feces from each animal were obtained and stored in cryovials at −80°C. On Days 0, 21, and 42 of the experiment, 1 mL of forelimb blood from each animal was collected and placed in a non‐anticoagulant tube, and the supernatant was separated by centrifugation after standing for 30 min, which was the serum sample. The basic diet composition was displayed in Table 1.

**TABLE 1:** Ingredient composition and nutrient levels of the basal diet for cats.

Items	Content
Ingredient, %	
Chicken powder	49.20
Chicken breast	7.37
Sweet potato granules	7.39
Beet pulp	6.08
Cassava starch	5.38
Chicken oil	8.14
Beef Powder	5.00
Rice	3.42
Beef tallow	2.15
Chicken liver powder	3.34
Salt	0.07
Yucca extract	0.10
Taurine	0.24
Choline chloride	0.24
Calcium hydrogen phosphate	1.06
Vitamin and mineral premix^a^	0.82
Nutrient composition^b^	
Metabolic energy, MC/kg	3.74
Dry matter, %	90.16
Crude fiber, %	2.02
Crude protein, %	36.99
Crude fat, %	20.16

^a^ The following was supplied by vitamin and mineral premix per kilogram of feed: vitamin A (15,000 IU), vitamin E (1800 IU), vitamin D_3_ (1000 IU), vitamin B_1_ (30.0 mg), vitamin B_2_ (28.0 mg), vitamin B_6_ (12.0 mg), vitamin B_12_ (1.1 mg), vitamin B_5_ (85.0 mg), vitamin B_3_ (110.0 mg), Fe (FeSO_4_) 50.0 mg, Ca (CaI_2_) 20.0 mg, Cu (CuSO_4_) 3.0 mg, Co (CoSO_4_) 0.1 mg, Mn (MnSO_4_) 18.0 mg, Zn (ZnSO_4_) 38.0 mg, Na (Na_2_SeO_3_) 0.1 mg, Se (Na_2_SeO_3_) 260.0 mg, I (CaI_2_) 40.0 mg.

^b^ The nutrient levels of the diet were analyzed.

During the experiment, each cat was housed individually. Cats were fed daily at 8:30 and 16:00 with the feeding amount meeting the energy requirements recommended by NRC (2006) for cats, and cats drank water freely. During the experiment, close attention was paid to cats' mental state, appetite, defecation status, and other noteworthy abnormalities. Cats were not scheduled to go outside except to perform the experiment. The animal rooms were kept well‐ventilated and clean, and a daily cleaning was conducted. Besides, the usual epidemic prevention standards were followed when performing the disinfecting operations.

#### 2.2.3 ELISA

Inflammatory cytokines, including TNF‐α, IL‐6, IL‐1β, and LPS, were measured by the commercial ELISA kits as directed by the manufacturer (Mlbio Co. LTD, Shanghai, China). Calprotectin and α1‐AT were also detected using the commercial ELISA kits. In addition, the contents of intestinal barrier proteins such as DAO, I‐FABP, and Zonulin were determined by ELISA kits. Among the above indicators, Zonulin, DAO, I‐FABP, LPS, IL‐6, TNF‐α, and IL‐1β were detected in serum. Calprotectin and α1‐AT were detected in feces.

#### 2.2.4 16S rRNA Sequencing

The 16S rRNA gene sequencing procedure was based on our earlier study, with slight modification (Liu et al. 2023). Briefly, following the extraction of the genomes of the fecal microbiota, the 16S rRNA genes of the V3 + V4 regions were amplified using the particular primers 341F (5′‐CCTAYGGGRBGCASCAG‐3′) and 806R (5′‐GGACTACNNGGGTATCTAAT‐3′). After being identified on a 2% agarose gel, the PCR products were purified, and the sequencing libraries were then created. Finally, the Illumina NovaSeq platform was used to sequence the library (Majorbio, Shanghai, China). The open‐source bioinformatics pipeline QIIME2 was used to analyze the raw data. Kruskal–Wallis, LEfSe, ANCOM, ANOVA, DESeq2, and other suitable techniques were applied to characterize bacteria with different abundances. The original data, which has the accession number PRJNA1242538, have been submitted to the NCBI SRA Database.

#### 2.2.5 SCFAs Detection

0.5 g of the fecal sample was dissolved in 10 mL of double‐distilled water. A 0.22 μm pore was utilized to filter the supernatant, which was produced after centrifuging at 15,000×*g* for 15 min at 4°C. The determination of SCFAs contents was performed by gas chromatography (Zhang et al. 2019). In brief, the capillary column was injected with a 1 μL aliquot. The carrier gas, nitrogen, had a flow rate of 2.0 mL/min. The injector and detector operated at a constant temperature of 240°C.

#### 2.2.6 Statistical Analysis

Data statistics were finished using SPSS software (IBM, Armonk, NY, United States). Normality and homogeneity of variance were assessed by Shapiro–Wilk and Levene's tests, respectively. Two‐way ANOVA was employed to analyze inflammatory cytokines and intestinal barrier proteins, followed by Tukey's HSD post hoc test (FDR‐adjusted *p* < 0.05). An independent sample t‐test was utilized to analyze SCFAs. The abundance of gut microbes was evaluated using the Kruskal–Wallis test (FDR‐adjusted *p* < 0.05). Microbial biomarkers were identified using LEfSe (LDA score > 2.0, *p* < 0.05 after FDR correction). Data were presented as mean ± standard error of mean (SEM). A significant difference is shown by a *p* < 0.05, a significant trend is indicated by a 0.05 ≤ *p* ≤ 0.10, and no significant difference is indicated by a *p* > 0.05.

## 3 Results

### 3.1 Effect of YCW on Intestinal Inflammation in Cats

**FIGURE 1:**
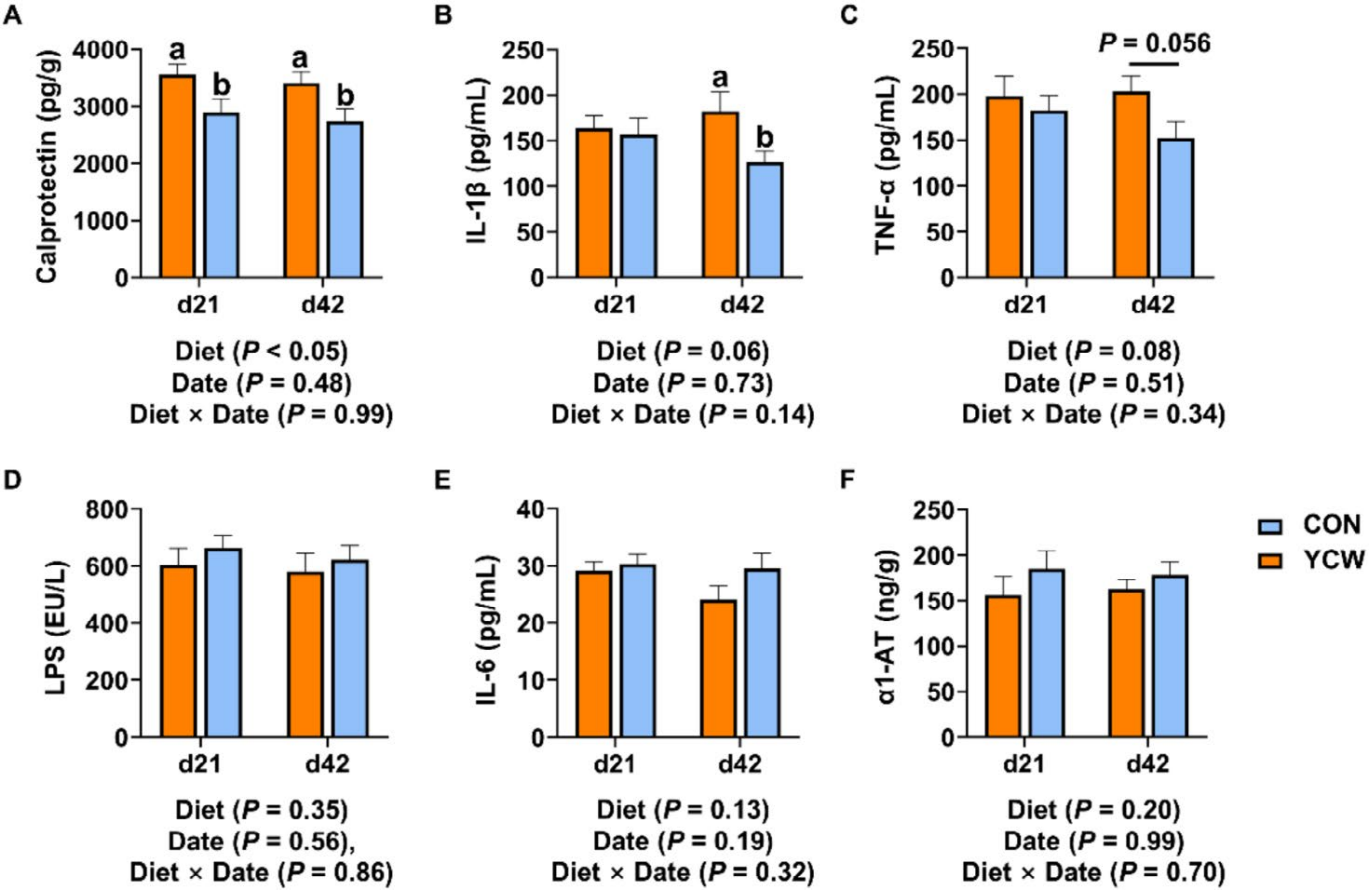
The contents of inflammatory factors in cats on Days 21 and 42 of the experiment. (A) Fecal Calprotectin. (B) Serum interleukin‐1β (IL‐1β). (C) Serum tumor necrosis factor‐α (TNF‐α). (D) Serum lipopolysaccharide (LPS). (E) Serum IL‐6. (F) Fecal alpha‐1 antitrypsin (α1‐AT). CON, cats fed with a basal diet; YCW, cats received the basal diet supplemented with 0.3% yeast cell wall. A significant difference between the CON and the YCW groups at the time point is indicated by values with an a, b (*p* < 0.05). A significant tendency is indicated when 0.05 ≤ *p* ≤ 0.10.

On Day 0 of the experiment, there was no noticeable distinction in any of the inflammatory markers between the control and the YCW groups, as indicated in Table 2 (*p* > 0.05). After cats ingested the YCW‐supplemented diet for 21 days, we found that the fecal Calprotectin content in the YCW group was markedly lower than that in the control group (*p* < 0.05) (Figure 1A). On Day 42 of the experiment, in contrast to the control group, the serum level of IL‐1β showed a significant decrease (*p* < 0.05) and the serum level of TNF‐α had a decreasing trend (0.05 ≤ *p* ≤ 0.10) in the YCW addition group (Figure 1B,C). In addition, the YCW group still had a lower fecal level of Calprotectin relative to the control group (*p* < 0.05) (Figure 1A). The fecal content of α1‐AT and the serum levels of IL‐6 and LPS did not differ significantly between the two groups (Figure 1D–F). These results suggested that YCW could ameliorate intestinal inflammation in cats to some extent.

**TABLE 2:** On Day 0 of the experiment, the contents of inflammatory factors and intestinal barrier proteins in cats.

Indicators	CON	YCW	SEM	*p*
LPS (EU/L)	668.49	729.28	27.04	0.272
IL‐1β (pg/mL)	169.24	159.65	15.46	0.766
TNF‐α (pg/mL)	175.67	206.55	16.13	0.352
IL‐6 (pg/mL)	28.11	31.49	2.15	0.446
Calprotectin (pg/g)	3652.1	3345.1	128.25	0.241
α1‐AT (ng/g)	185.35	206.41	10.79	0.343
DAO (ng/mL)	62.53	60.49	3.85	0.799
I‐FABP (pg/mL)	1703.5	1753.9	127.40	0.849
Zonulin (ng/mL)	412.35	536.01	43.43	0.16

*Note:* YCW, cats received the basal diet supplemented with 0.3% yeast cell wall.

Abbreviations: α1‐AT, alpha‐1 antitrypsin; CON, cats fed with a basal diet; DAO, diamine oxidase; I‐FABP, intestinal fatty acid binding protein; IL, interleukin; LPS, lipopolysaccharide; TNF‐α, tumor necrosis factor‐α.

### 3.2 Effect of YCW on the Intestinal Barrier Function in Cats

**FIGURE 2:**
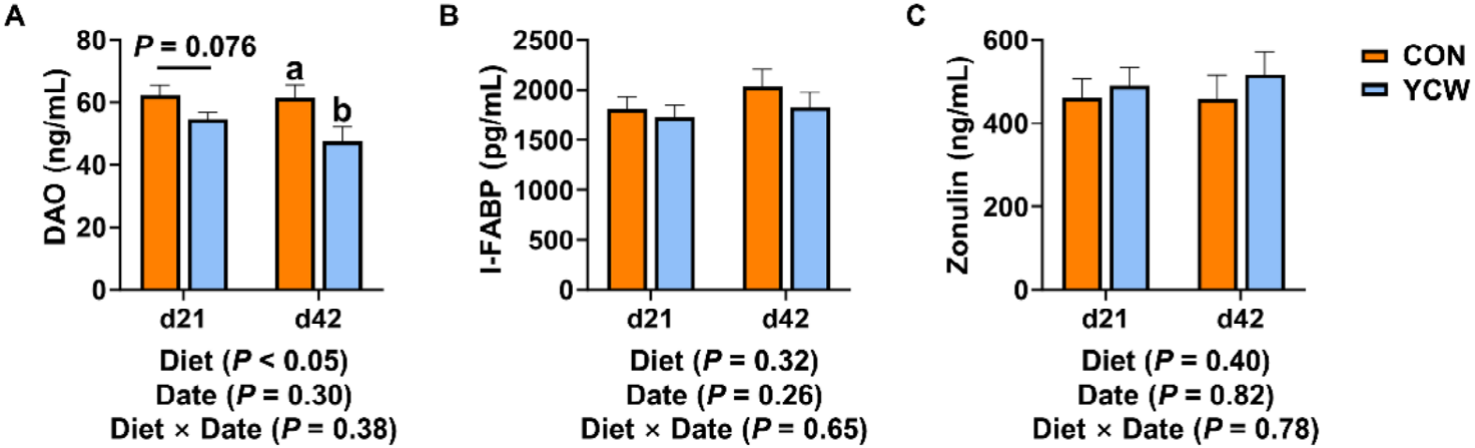
The contents of intestinal barrier proteins in cats on Days 21 and 42 of the experiment. (A) Serum diamine oxidase (DAO). (B) Serum intestinal fatty acid binding protein (I‐FABP). (C) Serum Zonulin. CON, cats fed with a basal diet; YCW, cats received the basal diet supplemented with 0.3% yeast cell wall. A significant difference between the CON and the YCW groups at the time point is indicated by values with an a, b (*p* < 0.05). A significant tendency is indicated when 0.05 ≤ *p* ≤ 0.10.

We also evaluated the impact of YCW on intestinal barrier function in cats. On Day 21 of the experiment, the level of serum DAO in the YCW group showed a declining trend in comparison to the control group (0.05 ≤ *p* ≤ 0.10) (Figure 2A). After 42 days of supplementation with YCW, the YCW group had a significantly decreased level of DAO in serum when compared with the control group (*p* < 0.05) (Figure 2A). Other intestinal barrier proteins, including I‐FABP and Zonulin, did not markedly alter between the control and the YCW groups (*p* > 0.05) (Figure 2B,C).

### 3.3 Effect of YCW on Gut Microbiota Composition in Cats

**FIGURE 3:**
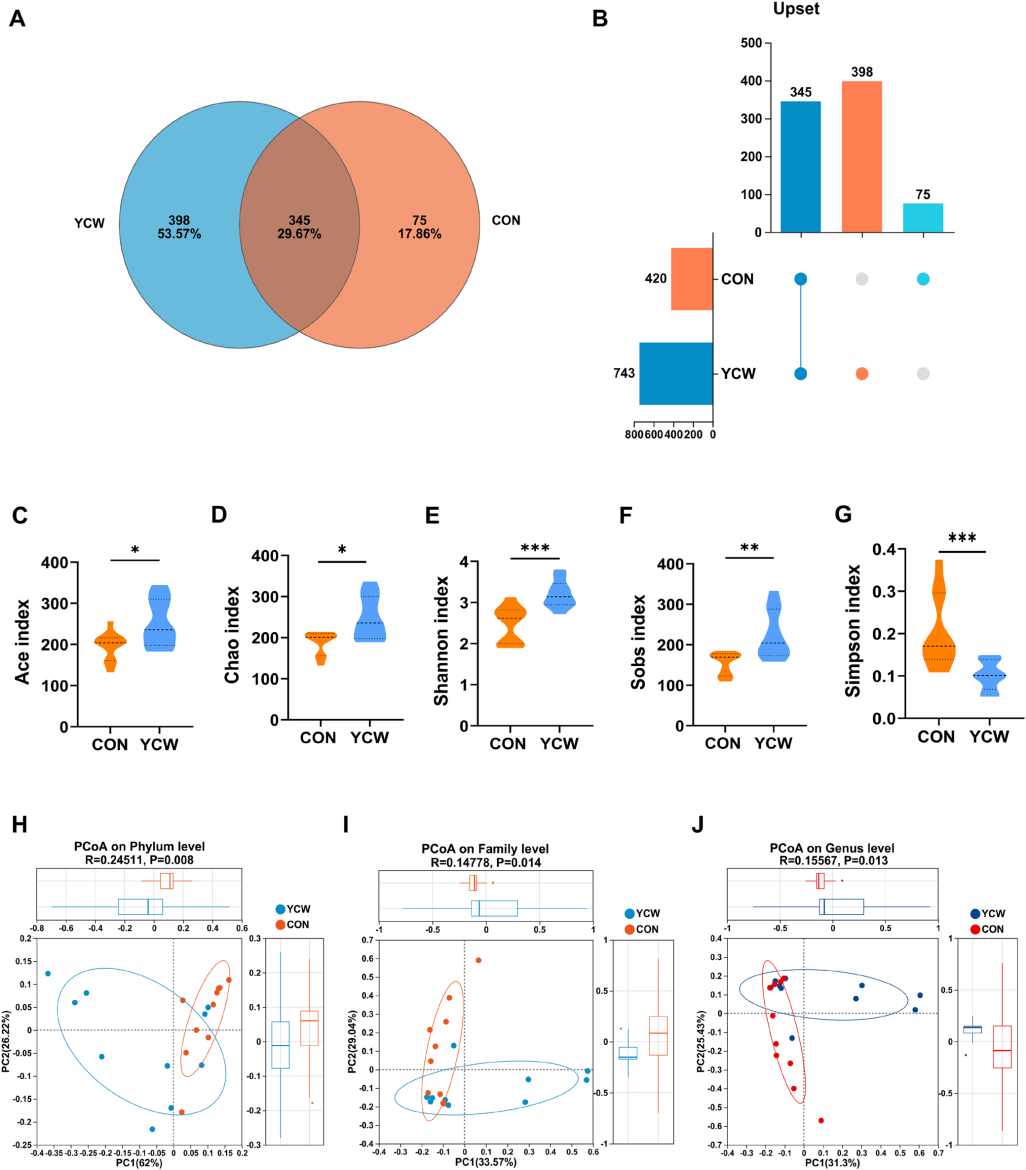
Analysis of microbial species structure in cats on Day 42 of the experiment. (A) The Venn diagram. (B) The Upset diagram. (C–G) The Ace, Chao, Shannon, Sobs, and Simpson indexes. (H–J) The PCoA analysis on the phylum, family, and genus levels. CON, cats fed with a basal diet; YCW, cats received the basal diet supplemented with 0.3% yeast cell wall. ***p ≤ 0.001, **0.001 < *p* ≤ 0.01, *0.01 < *p* < 0.05.

We next analyzed whether the addition of YCW affected the intestinal microbiota composition in cats. As exhibited in Figure 3A,B, a total of 818 OTUs were identified in the two groups, of which 75 and 398 specific OTUs were observed in the control and the YCW groups, respectively. The richness and diversity of microbial communities between the two groups were evaluated using alpha diversity analysis. The Ace, Chao, Shannon, and Sobs indices all dramatically rose, whereas the Simpson index dropped in the YCW group in comparison with the control group (*p* < 0.05) (Figure 3C–G). To ascertain how the two groups' communities were distributed differently, a beta diversity analysis was further performed. The PCoA plots on phylum, family, and genus levels all showed that the clouds derived from the control and the YCW groups were distinctly separated from each other (Figure 3H–J).

**FIGURE 4:**
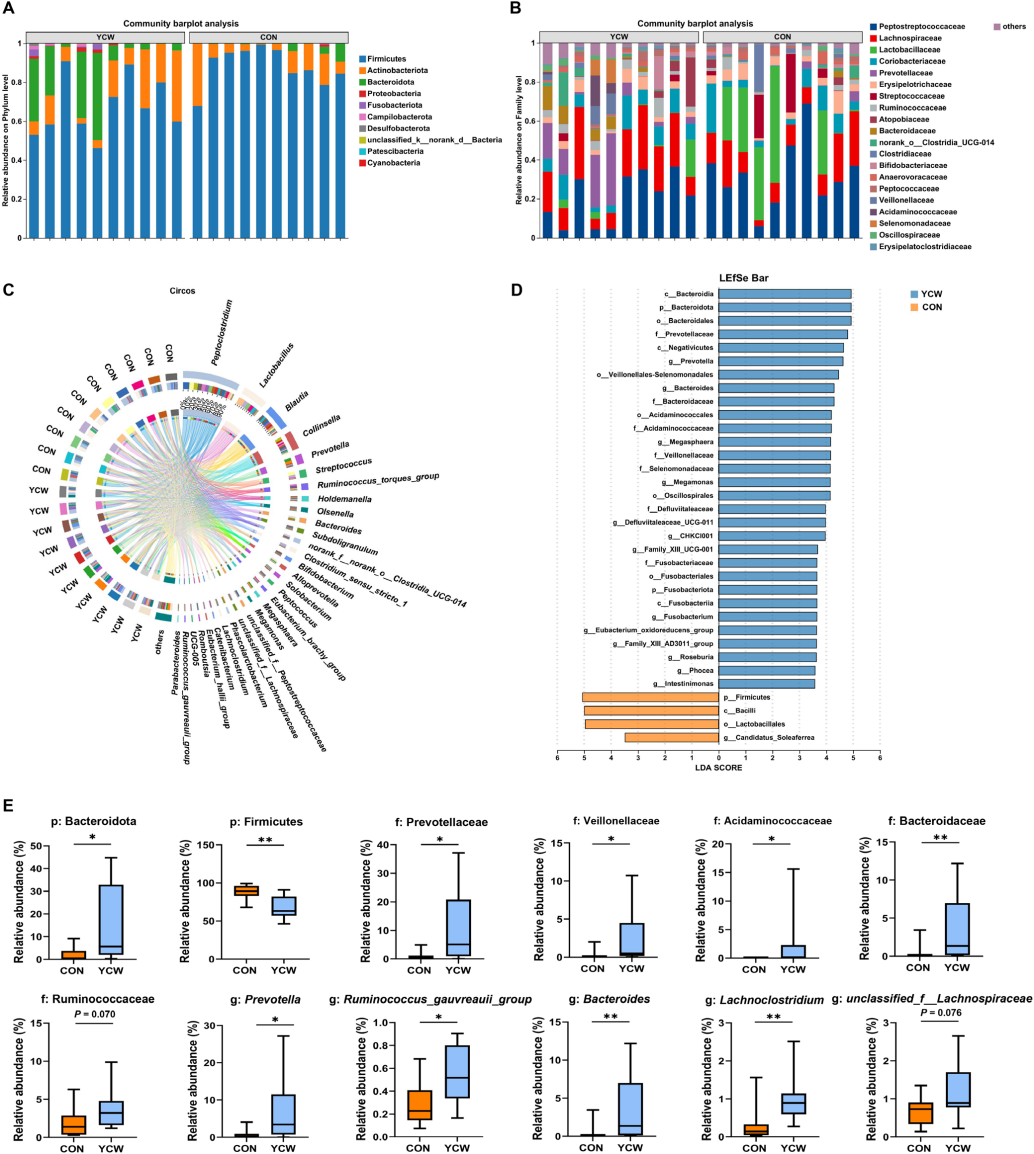
The difference in the intestinal microbiota composition between the two groups on Day 42 of the experiment. (A) The phylum‐level community barplot analysis. (B) The family‐level community barplot analysis. (C) The community Circos diagram at the genus level. (D) The LEfSe analysis. (E) The percentage of the fecal microbiota on the phylum, family, and genus levels. CON, cats fed with a basal diet; YCW, cats received the basal diet supplemented with 0.3% yeast cell wall. **0.001 < *p* ≤ 0.01, *0.01 < *p* < 0.05.

At the bacterial phylum, the YCW group had a greater abundance of Bacteroidota and Proteobacteria and a lower abundance of Firmicutes than the control group (*p* < 0.05) (Figure 4A,E). At the bacterial family level, relative to the control group, YCW consumption significantly increased the relative abundance of Prevotellaceae, Veillonellaceae, Acidaminococcaceae, and Selenomonadaceae (*p* < 0.05) (Figure 4B,E). In addition, Bacteroidaceae were more abundant in cats in the YCW group (*p* < 0.05) (Figure 4B,E). In contrast to the control group, the abundance of Ruminococcaceae had an upward trend, whereas the abundance of Peptostreptococcaceae and Lactobacillaceae showed a decreasing trend in the YCW group (0.05 ≤ *p* < 0.10) (Figure 4B,E). The feline intestinal bacteria with abundance in the top 30 genera were also analyzed; the proportions of *Prevotella*, *Bacteroides*, *Lachnoclostridium*, and *Ruminococcus_gauvreauii_group* were higher in cats in the YCW addition group than those in the control group (*p* < 0.05) (Figure 4C,E). Furthermore, *unclassified_f__Lachnospiraceae* showed an enrichment trend in the YCW group (0.05 ≤ *p* < 0.10) (Figure 4C,E). On the contrary, *Peptoclostridium* and *Lactobacillus* both showed an enrichment trend in the control group (0.05 ≤ *p* < 0.10) (Figure 4C,E). The result of the linear discriminant analysis supported the preceding findings (Figure 4D).

### 3.4 Effect of YCW on SCFAs Production in Cats

**FIGURE 5:**
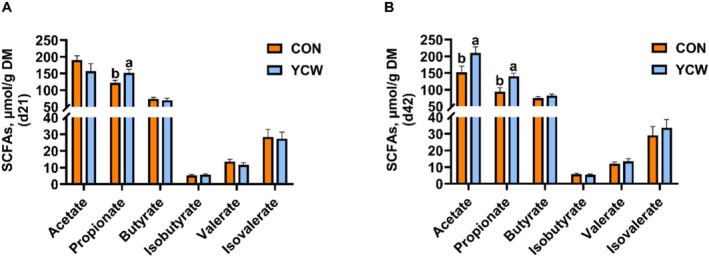
The SCFAs contents in cats on Days 21 and 42 of the experiment. (A) The levels of acetate, propionate, butyrate, isobutyrate, valerate, and isovalerate in cats on Day 21. (B) The SCFAs levels in cats on Day 42. CON, cats fed with a basal diet; YCW, cats received the basal diet supplemented with 0.3% yeast cell wall. A significant difference between the CON and the YCW groups at the time point is indicated by values with an a, b (*p* < 0.05).

Since SCFAs are important metabolites of gut microbes, we next examined the SCFAs concentrations in cats. On Day 21 of the experiment, we found that cats in the YCW group had an increased propionate level when compared with cats in the control group (*p* < 0.05) (Figure 5A). On Day 42 of the experiment, the fecal levels of acetate and propionate were markedly enhanced in the YCW group relative to the control group (*p* < 0.05) (Figure 5B). The two groups did not differ in their levels of butyrate, isobutyrate, valerate, or isovalerate (*p* > 0.05) (Figure 5A,B).

## 4 Discussion

As a prebiotic composed primarily of α‐D‐mannan and β‐D‐glucan, YCW can boost digestive tract homeostasis by establishing favorable environments for beneficial bacteria. YCW and its components have been used in economic animal breeding and human functional food and have biological activities such as improving intestinal morphology and barrier integrity, changing intestinal flora structure, and enhancing immune capacity (Liu, Wang, et al. 2021; Liu, Wu, et al. 2021). Along with the growing number of pets and the global trend for pet owners to regard pets as family members, the health of dogs and cats has received widespread attention. As a natural carnivore, the gut microbiota structure of cats is different from that of omnivores and herbivores, and dietary modifications to the gut microbiota and its metabolites may have an impact on the overall health of felines. However, the effects of adding YCW to diets on intestinal health in cats have not been systematically and adequately studied. Thus, the purpose of this study was to look at how dietary YCW supplementation affected gut microbial structure, metabolites, and gut health in cats. The analysis of the related mechanism of YCW action can help to broaden the understanding of the unique nutritional and metabolic requirements of cats as carnivores and offer a theoretical basis for promoting the development of novel functional foods and precision nutrition for cats.

Bacteroidota is one of the key regulators of homeostasis in the body and is involved in both intestinal and extra‐intestinal roles by influencing numerous physiological processes such as metabolism, maintenance of barrier integrity, inflammatory regulation, and hematopoiesis (Yatsunenko et al. 2012). The abundance of Bacteroidota species was lower during Crohn disease (CD), and it is reported that a subgroup of patients with Ulcerative colitis (UC) had less than 1% of Bacteroidota in their gut microbiota (Brown et al. 2019). In our study, the contents of Bacteroidota and its members, including Bacteroidaceae and *Bacteroides*, were all significantly enhanced in cats fed YCW. Members of the family Ruminococcaceae decline, are the most frequent microbial alterations linked to intestinal inflammation. Ruminococcaceae genera have anti‐inflammatory and intestinal barrier‐protective properties (Xue et al. 2022). Compared to dogs with intestinal diseases, healthy individuals had higher abundance of Ruminococcaceae (Alessandri et al. 2020). Our results revealed that YCW supplementation significantly elevated the relative contents of Ruminococcaceae and *Ruminococcus_gauvreauii_group* in cats, which further indicated that YCW improved the host gut microbiota structure. *Prevotella* is a genus with well‐established dual roles in gut health. On the one hand, it belongs to the probiotics genus and is known to boost glucose metabolism and prebiotic consumption (Kovatcheva‐Datchary et al. 2015). A study has shown that individuals with inflammatory bowel disease (IBD) had far lower levels of *Prevotella* in their gut (Han et al. 2024), suggesting a potential protective role in maintaining intestinal homeostasis. In the current investigation, we observed that YCW increased the percentage of *Prevotella* in feline feces, which may contribute to the observed improvements in intestinal health. On the other hand, however, accumulating evidence highlights its pro‐inflammatory potential. Recent research has indicated a possible connection between *Prevotella* and inflammatory and immunological disorders (Alpizar‐Rodriguez et al. 2019; Iljazovic et al. 2021). A prior study found that the enhanced *Prevotella* abundance was linked to the anabatic mucosal inflammation mediated by T helper type 17 (Th17) (Larsen 2017). This duality might be attributed to strain‐specific differences: although some *Prevotella* strains produce anti‐inflammatory metabolites such as SCFAs, others may secrete pro‐inflammatory factors or disrupt the intestinal barrier when overgrown, especially in the context of dysbiosis. Additionally, the host's physiological state and dietary context likely modulate *Prevotella's* functional outcome. Its beneficial effects may predominate in a balanced gut ecosystem, whereas its pro‐inflammatory properties may be amplified under perturbed conditions (Abdelsalam et al. 2023; Tett et al. 2021). In subsequent studies, metagenomic sequencing will help to further explore the specific strains that play particular roles. In addition, the enrichment of *Lachnoclostridium* was noted in cats in the YCW group. The recently identified genus *Lachnoclostridium* is considered a beneficial group of gut flora within the extremely polyphyletic class Clostridia. *Lachnoclostridium* has an anti‐inflammatory impact and is crucial for intestinal homeostasis (Song et al. 2023). Our findings corroborate previous studies on cats supplemented with varying doses of YCW (González et al. 2023), demonstrating a significant increase in the relative abundance of Bacteroidota and *Prevotella*.

Since metabolites produced by bacteria are important for host health, it is imperative to clarify the metabolic effects of gastrointestinal homeostasis. SCFAs are organic fatty acids made up of 1–6 carbon atoms, which are significant products of intestinal flora. SCFAs are mainly produced by the colonic microbial fermentation of dietary carbohydrates, including acetate, propionate, and butyrate. Microorganisms in the gut hydrolyze carbohydrates to monosaccharides, which are subsequently fermented to phosphoenolpyruvate via the Embden‐Meyerhof pathway, and phosphoenolpyruvate intermediates are then created through different reactions to form SCFAs (Koh et al. 2016). In this investigation, the addition of YCW markedly raised the propionate content on Day 21 and the acetate and propionate contents on Day 42, which was in line with the results of the microbial alterations. The increased propionate may be a result of the dramatically higher abundance of Bacteroidota and its members, including Bacteroidaceae and *Bacteroides*. Dietary carbohydrates driven by the Bacteroidota are the main source of propionate production, and it has been shown that the amount of propionate in human feces was correlated with the relative abundance of Bacteroidota (Louis and Flint 2017). Species within the Lachnospiraceae and Ruminococcaceae families are known to break down undigested carbohydrates, generating SCFAs such as acetate and propionate as their major fermentation end products (Charalambous et al. 2025). According to a previous report, bacteria belonging to the Ruminococcaceae family were positively associated with the plasma acetate level (Moreno‐Navarrete et al. 2018). Another study revealed that *Lachnoclostridium* could suppress pro‐inflammatory responses via acetate production (Liu et al. 2025). Thus, the enhanced concentrations of acetate and propionate in cats might be attributed to a great improvement in the abundance of acetate‐ or propionate‐producing bacteria induced by YCW.

Many intestinal anaerobic bacteria can generate acetate via acetyl‐CoA or the Wood‐Ljungdahl route. Acetate is the principal metabolite that the majority of anaerobic bacteria in the colon make in order to ferment undigested and unabsorbed carbohydrates in the small intestine. It is also the primary substrate for the synthesis of cholesterol, which is the main source of energy that bacteria use to supply the host (Ballard 1972). Propionate can be produced by succinic acid, acrylate, or propylene glycol pathways. After absorption by the colon, propionate enters the liver through the portal vein and is subsequently metabolized by liver cells to participate in the process of reversal of pyruvate into glucose (Harrison 1992). A study using a reactor system that mimics the large intestine environment and enables maintaining stable bacterial communities has found that the addition of brewer's YCW dramatically increased the amounts of acetate and propionate induced by *Megasphaella* and *Veillonella* (Nakashimada et al. 2011). Another study assessed the effects of *Saccharomyces cerevisiae* products composed of 22.5% MOS and 27.5% β‐glucan on the gut flora and its fermentation metabolite composition in the canine gastrointestinal tract simulated in vitro, and found that propionate production was increased in the proximal and distal colon and the alterations in fermentation metabolites might be related to specific propionate‐producing microbial changes, such as Prevotellaceae and Porphyromonadaceae (Van den Abbeele et al. 2020). It is worth noting that research has evaluated the in vitro and in vivo fecal microbiota and fermentation responses in cats consuming diets with different doses of YCW and found that the low and high doses of YCW both could modify fecal SCFAs generation, including propionate, acetate, lactate, and valerate, but only a high dose of YCW raised the butyrate content (González et al. 2023). Butyrate, which is usually formed by the condensation of two acetyl‐CoA molecules via butyrate kinase or butyryl‐CoA transferase, is a key substance that promotes intestinal integrity and provides the primary energy requirements for intestinal epithelial cell proliferation and development. Nonetheless, no significant change in butyrate level was found in our study, which may be related to the amount of YCW added.

SCFAs have been stated to be essential for preserving normal intestinal morphology and mending the integrity of the intestinal epithelial barrier, but most of these studies focused on butyrate and indicated positive effects of butyrate on damaged intestinal epithelial repair and barrier function (Bach Knudsen et al. 2018). On the other hand, however, butyrate has been demonstrated to be toxic to colonic epithelial cells in vitro, particularly when the intestinal mucous layer is changed (Rhodes 2021). Although less studied than the effects of butyrate on the intestinal barrier, propionate and acetate are also of interest and have demonstrated certain protective properties in the gut, such as stimulating butyrate‐producing bacteria by cross‐feeding and having less harmful effects on epithelial cells (Liu, Wang, et al. 2021; Liu, Wu, et al. 2021). On the basis of one study, acetate administration may have an anti‐inflammatory influence and promote the expression of the barrier gene in the UC‐derived epithelial cell model (Deleu et al. 2023). Research revealed that acetate could specifically bind to free fatty acid receptor 2 and suppress NF‐κB‐MLCK‐MLC signaling, which further re‐establishes the intestinal epithelium integrity and increases the expression of tight junctional proteins (Song et al. 2024). In addition, a previous study displayed that intestinal tight junction‐associated protein endothelial cell‐selective adhesion molecule was up‐regulated by the propionate treatment in DNA microarray analysis, which demonstrated how propionate enhanced the intestinal barrier function (Isayama et al. 2023). In this research, we examined the serum indicators of the intestinal barrier, including LPS, DAO, I‐FABP, and Zonulin, and found that cats consuming the diet with YCW had a decreased serum DAO level on Day 42 compared to cats in the CON group. Serum DAO is a perfect indication for assessing intestinal barrier impairment since it has a positive correlation with the permeability of the intestinal barrier (Wang et al. 2020). In broilers with necrotic enteritis, dietary YCW was found to increase the populations of Bifidobacteria and *Lactobacilli* while lowering the blood levels of DAO and LPS (Liu et al. 2018). In addition, dietary treatment with β‐glucan, the primary element of YCW, significantly elevated the promotion of the zonula occludens in the jejunal epithelium and reduced the serum contents of DAO and D‐lactate in weaned pigs orally infused with enterotoxigenic *Escherichia coli* (Zhou et al. 2022). Therefore, these findings revealed that the supplementation of YCW in the diet may improve intestinal health in cats by reducing DAO levels, which in turn effectively decreased intestinal permeability and enhanced the intestinal barrier function. Nevertheless, YCW showed no significant effects on other serum intestinal barrier indicators, such as I‐FABP and Zonulin, in this study. I‐FABP is specifically expressed on the epithelial cells of the mammalian small intestine and is a constitutively expressed intracellular protein that is only released after cell necrosis (Schellekens et al. 2017). Therefore, the more severe the intestinal mucosa injury is, the more I‐FABP is released into the blood. If the injury does not reach the level of necrosis, then the I‐FABP content may remain unchanged. Zonulin correlates with intercellular junctions and may not directly connect with the severity or type of physical barrier disruption, and our results suggested that intestinal epithelial tight junctions were not significantly altered in cats. In addition, why these intestinal barrier indicators were not changed may be related to the sensitivity threshold of these biomarkers and the treatment duration of YCW. Future studies could extend the intervention period and combine intestinal histology validation to comprehensively identify the multi‐target effects of YCW.

There is a close and bidirectional relationship between the intestinal barrier and intestinal inflammation. The two influence each other and form a vicious cycle, especially in animals prone to chronic intestinal diseases, such as cats. Pro‐inflammatory factors such as TNF‐α can be released when the intestinal physical barrier is disrupted or the immune barrier is dysregulated. Barrier damage will be further exacerbated by inflammatory mediators and infiltration of inflammatory‐related cells (Okumura and Takeda 2024). By measuring the contents of inflammatory factors, we found that cats consuming YCW had a lower fecal Calprotectin level than cats in the control group. Calprotectin is described as a calcium‐binding and antimicrobial protein that is generated from the plasma of neutrophils in the inflammatory process and has been a promising indicator of intestinal inflammation (González et al. 2024). A study has found a difference in the concentration of fecal Calprotectin between dogs with chronic diarrhea and normal dogs, and the concentration of Calprotectin in the feces of normal dogs was lower than that of dogs with chronic diarrhea linked to histologic lesions (Grellet et al. 2013). In addition, a study involving cats revealed that healthy cats had a lower fecal Calprotectin content than cats with small cell intestinal lymphoma or chronic inflammatory enteropathies, which confirmed that intestinal Calprotectin was involved in the pathogenesis of chronic enteropathies in felines (Riggers et al. 2023). In our study, cats also showed decreased serum levels of IL‐1β and TNF‐α after YCW intake. TNF‐α is an inflammatory cytokine secreted by monocytes and macrophages during acute inflammation and is in charge of a variety of signaling processes inside cells that result in necrosis or apoptosis (Balkwill 2006). IL‐1β is also a master regulator of pro‐inflammation and is induced by inflammatory signals in a broad number of immune cell types. In agreement with our findings, it is illustrated that YCW considerably reduced the serum levels of TNF‐α and IL‐1β in weaned pigs (Lee et al. 2021). These findings suggested that dietary YCW has some anti‐inflammatory benefits on cats. In addition, we also detected other inflammatory cytokines, including IL‐6 and α1‐AT, which did not differ significantly between the control and the YCW groups. It is reported that TNF‐α and IL‐1β are early pro‐inflammatory cytokines and are the first to be released in inflammatory responses. In contrast, IL‐6 is a secondary inflammatory mediator, and its release occurs relatively late (Wang et al. 2024). α1‐AT is an acute‐phase protein, more reflective of systemic inflammation, and requires a strong enough stimulus to trigger synthesis by hepatocytes (Frangolias et al. 2003). Thus, IL‐6 and α1‐AT may not undergo significant level alterations when inflammation is not severe enough.

On the basis of all the above results, we have demonstrated that dietary YCW improved the intestinal health of cats by regulating the intestinal microbiota‐metabolites‐intestinal barrier axis. YCW significantly modulated the structure and composition of the intestinal flora in cats, increasing the populations of acid‐producing and propionate‐producing bacteria and raising the concentrations of acetate and propionate, which in turn promoted the intestinal barrier function and reduced the inflammatory level.

## 5 Conclusion

In summary, incorporating YCW into the food of cats improved the intestinal barrier function and alleviated intestinal inflammation. Furthermore, dietary YCW increased the percentage of Bacteroidaceae, Ruminococcaceae, *Blautia*, and *Lachnoclostridium*, and elevated the contents of metabolites such as acetate and propionate, which might be responsible for the greater intestinal barrier and immunity function. These findings give the use of YCW in pet food to enhance the intestinal health and well‐being of companion animals scientific legitimacy.

## Author Contributions

**Xiaoxi Liu:** formal analysis (equal), investigation (equal), visualization (equal). **Haotian Wang:** data curation (equal), methodology (equal). **Yuqing Lu:** methodology (equal), supervision (equal). **Guoqing Jin:** resources (equal), validation (equal). **Le Fu:** resources (equal), validation (equal). **Hongxia Mao:** resources (equal), validation (equal). **Yi Wu:** funding acquisition (equal), supervision (equal), writing – review and editing (equal). **Pan Liu:** methodology (equal), software (equal), visualization (equal), writing – original draft (equal), writing – review and editing (equal).

## Conflicts of Interest

The authors declare no conflicts of interest.

## Supporting information


**Table S1:** Analysis of the main components of YCW.

## Data Availability

The corresponding author will provide the data supporting the study's conclusions upon reasonable request.
