# Supplementary material for: Dietary Supplementation With Yeast Cell Wall Modulates Gut Microbiota and SCFAs Production to Improve Intestinal Health in Adult Cats
Source: Food Sci Nutr. 2025 Sep 26;13(10):e71007. doi: 10.1002/fsn3.71007 (PMC12464450; doi:10.1002/fsn3.71007)
Supplement: Supplementary file 1 — Table S1: Analysis of the main components of YCW. [file FSN3-13-e71007-s001.docx]

Supplementary Table 1 Analysis of the main components of YCW.

| Items, % | Content | Method |
| --- | --- | --- |
| Moisture | 6.10 | GB/T 6435-2014 8.1 |
| Crude protein | 25.38 | GB/T 6432-2018 7.2 |
| α-Mannan | 24.20 | QB/T 4572-2021 6.4.1 |
| β-Glucan | 31.10 | QB/T 4572-2021 6.4.1 |
